# 

**Published:** 1898-06

**Authors:** 


### CONTENTS.

ORIGINAL COMMUNICATIONS. PAGE

The Dawn of Modern Dental Literature. By WILLIAM H. TRUEMAN, D.D.S., Philadelphia . . . . . . 333

The Amalgam Question and Dentists with "Yankee Wit." By CHARLES H. TAFT, A.B., D.M.D., Boston, Mass. . . . . . . 344

Serration an Error in Practice. By J. W. WORCESTER, M.D., Middletown, N. Y. 355

Studies in Evolution. By EUGENE S. TALBOT, M.D., D.D.S., Chicago, Ill. . . . 357

ABSTRACTS AND TRANSLATIONS.

Practical and Theoretical Trials of "Formagen." By MAX BAUCHWITZ, Stettin, Prussia . . . . . . 358

REPORTS OF SOCIETY MEETINGS.

American Academy of Dental Science . . . . . . 362

Academy of Stomatology . . . . . . 383

Odontological Society of Pennsylvania . . . . . . 389

EDITORIAL.

The Future Entrance Standard . . . . . . 397

BIBLIOGRAPHY . . . . . . 401

DOMESTIC CORRESPONDENCE.

A Reply to the Editor's Query . . . . . . 403

NOTES AND COMMENTS . . . . . . 404

CURRENT NEWS.

The National Dental Association . . . . . . 409

American Medical Association, Section on Stomatology . . . . . . 410

New Jersey State Dental Society . . . . . . 410

New Jersey Summer Examinations . . . . . . 411

Missouri State Dental Association . . . . . . 411

Colorado State Dental Association . . . . . . 412

Pennsylvania State Dental Society . . . . . . 412

